# Validation of the Assessments of Adult Health Literacy: A Rasch Measurement Model Approach

**DOI:** 10.21203/rs.3.rs-3164944/v1

**Published:** 2023-08-09

**Authors:** Sasha A. Fleary

**Affiliations:** CUNY Graduate School of Public Health and Health Policy

**Keywords:** health literacy, measurement, health communication, functional health literacy, interactive health literacy, critical health literacy, communicative health literacy

## Abstract

**Background:**

Both the World Health Organization and U.S. Department for Health and Human Services have emphasized the importance of health literacy (HL) to improving population health and reducing health disparities. HL includes three core areas/qualities: functional (i.e., health-related reading, writing, and numeracy), interactive/communicative (i.e., skills for interacting with multiple constituents and sources of information and navigating the health environment), and critical (i.e., personal and community advocacy for health). HL is implicated in medical adherence, preventive health, mental health stigma and help-seeking, and health decision-making. Though HL is critical to health and health decision-making, research on HL is still relatively limited, with most research focusing on functional HL. A major gap in research is related to the lack of measurement of interactive and critical HL. To address this gap, this study modified and assessed the validity of the Assessments of Adolescent Health Literacy (AAHL-Adolescent), test-based assessments of adolescents’ functional, interactive, and critical HL, in an adult sample.

**Methods:**

One item from the AAHL-Adolescent item bank was modified to be more appropriate for an adult sample. Adults (n=2346) completed a measurement battery that included the HL item bank (12 functional, 15 interactive, and 9 critical HL questions), Newest Vital Sign (NVS), Single-Item Literacy Scale (SILS), demographics, and questions about HL-related behaviors. The assessments were evaluated and validated using Rasch measurement models. Convergent and criterion validity were assessed.

**Results:**

The final 7-item functional, 10-item interactive, and 7-item critical HL assessments and their composite (24 items) fit their respective Rasch models. Item-level invariance was established for gender, ethnicity, education, and age across all assessments. Differential item functioning for race was noted for two items on the interactive HL assessments. Good convergent validity with the NVS and SILS and good criterion validity with the HL-related behaviors were observed for all assessments.

**Conclusions:**

The AAHL-Adult is the first test-based instrument validated in the U.S. that includes assessments for all three core qualities of HL. These assessments have utility across multiple settings, including public health program planning and evaluation, intervention development and evaluation, and clinical settings.

It is estimated that only 12% of US adults have proficient health literacy [HL](1). HL “*entails people’s knowledge, motivation and competencies to access, understand, appraise, and apply health information in order to make judgments and make decisions in everyday life concerning healthcare, disease prevention, and health promotion to maintain or improve quality of life during the life course*” (2), p. 3. It is both a social determinant of health and a skillset that can mitigate the effects of other social determinants of health (3–5). HL is implicated in medical adherence (6), preventive health (7), mental health stigma and help-seeking (8), and parents’ and adolescents’ health behaviors (9). Both the World Health Organization (10) and the US Department for Health and Human Services (11) have emphasized the importance of HL to improving population health and reducing health disparities.

Though the concept has increased in visibility and is critical to health and health decision-making across the lifespan, research on HL is still relatively limited. Sorensen and colleagues (2) argued that HL can be divided into core concepts and applied use. Core concepts include Nutbeam’s (12) functional HL (i.e., health-related reading, writing, and numeracy), interactive/communicative HL (i.e., skills for interacting with multiple constituents and sources of information and navigating the health environment), and critical HL (i.e., personal and community advocacy for health). Applied use is how a person uses HL in multiple roles, such as a patient navigating health care or a consumer purchasing health products. Most of the existing research on HL has focused on functional HL, likely due to the lack of measurement of the other core qualities of HL.

A scan of the 198 adult HL measures listed in the Boston University’s Health Literacy Tool Shed database (13) found over 100 unique adult HL measures (excluding translations and shortened/extended versions of existing measures) as of April 2023. Of these unique measures, about 50 were test-based, with 26 measuring functional HL and 1 measuring both interactive and critical HL. Over 50 unique measures were perceptions-based, inquiring about individuals’ perceived competencies for using HL skills. Over 60 measures were disease- or behavior-specific; however, this included multiple versions (e.g., language translation, short form) of the measures. A glaring limitation of these measures is that the Korean Health Literacy Instrument (14) was the only test-based measure of individuals’ interactive and critical HL skills. This instrument has only been validated in a Korean sample.

In a study using perception-based measures, Fleary and colleagues (8) found negative relationships between functional and interactive/communicative HL and mental health stigma as well as aversion to mental health help-seeking. They also found that adults with higher interactive/communicative HL were more willing to interact with people with mental illnesses. Further, Joseph and colleagues (15) found that interactive/communicative and critical HL moderated the relationship between food insecurity and emotional eating in unexpected ways. At higher levels of food insecurity, higher interactive/communicative and critical HL was associated with higher emotional eating. In adolescent research using test-based measures of interactive and critical HL, Fleary and colleagues (16) found that interactive and critical HL were differentially related to factors affecting adolescents’ substance use behaviors (e.g., both interactive and critical HL were related to risk perception and self-efficacy for substance use but only functional and interactive HL were related to adolescents’ substance use behaviors). The extant literature on the effect of functional HL on health behaviors and outcomes (17–20) suggests that functional HL interventions may be critical to improving health outcomes and addressing health disparities. However, the lack of interactive and critical HL measures, especially test-based measures, limits inferences about these types of HL and likely results in important core aspects of HL not being considered in interventions, health communications, or clinical encounters.

## Current Study

Given the lack of test-based assessments for interactive and critical HL, this study aimed to assess the validity of the Assessments of Adolescent Health Literacy (AAHL-Adolescent) in an adult sample. The AAHL-Adolescent includes test-based assessments of functional, interactive, and critical HL developed and validated in an adolescent sample using Rasch Measurement Models (21). The Rasch measurement model is a probabilistic model that applies sample-independent methodology by fitting data to a measurement model rather than to a sample, which is done in classical test theory (22). An important consideration of the Rasch measurement model is that item difficulty and person ability are measured independent of the sample and items in the measure, respectively (23). The methodology involves calculating the probability of a person responding in a specific manner to a specific item with the assumption that persons with higher ability will have higher probabilities of correctly endorsing the items, and items with lower probabilities of being correctly endorsed are those with higher difficulties. For the current study, minor changes were made to the wording of some items in the AAHL-Adolescent to make it more applicable to adults. The Rasch measurement model and other reliability and validity measures were used to assess the utility of the adapted AAHL-Adolescent for adults.

## Methods

### Study Design

This study assessed the psychometric properties of the Assessments of Adult Health Literacy (AAHL-Adult), an adult version of the AAHL-Adolescent (21). This study was approved by the Tufts University Social Behavioral and Educational Institutional Review Board. Participants provided consent prior to completing the assessment battery.

### Sample and Data Collection

Data were collected as part of a study of parent-adolescent dyads’ HL and health behaviors. Data were collected from 2346 parents between February and October 2020 using the Qualtrics survey panel. Qualtrics randomly selected a sample that was 45% male, 50% with less than a college degree, 40% income >$100,000, and predominantly racial and ethnic minorities (i.e., no more than 35% White). These quotas ensured meaningful comparisons could be made for groups that tend to be underrepresented in research. Qualtrics’ recruitment included sending emails to a stratified (based on aforementioned characteristics) random sample of panelists who were 18 years or older. Panelists who opted to participate in the study first saw a screen with the consent form and were directed to the survey once they consented to participate. If they did not agree to participate, they were thanked for their time and did not see any further information. The survey included questions about HL and health behaviors. Attention check questions were also included to ensure participants were attentive throughout the survey. The survey took about 20 minutes to complete, and participants chose incentives determined by Qualtrics (e.g., airline miles, gift cards, redeemable points).

### Measures

#### Demographics

Participants self-reported their demographic characteristics. Age was reported in years. Participants selected their gender from six options: male, female, transgender (male to female), transgender (female to male), transgender (gender non-conforming), and other. Participants indicated whether they were of Hispanic, Latino/a, or Spanish origin. They also selected their race from the following options: American Indian or Alaskan Native, Asian, Black or African American, Native Hawaiian or Other Pacific Islander, White, multiple races, and prefer not to answer. Participants indicated their highest level of education completed from the following options: less than high school, high school graduate or equivalent, some college, Associate’s degree (including occupational or academic degrees), Bachelor’s degree, and graduate degree. Questions on gender, race, ethnicity, education, and income also included ‘prefer not to answer’ as an option.

#### Assessments of Adult Health Literacy (AAHL-Adult)

The AAHL-Adult is the adult version of the AAHL-Adolescent and includes three test-based HL assessments that align with Nutbeam’s (12) definitions of functional, interactive/communicative, and critical HL. The AAHL-Adolescent was developed using a multi-phase mixed methods design, and the questionnaire development process is summarized here and reported in detail elsewhere (21). The initial item bank for the AAHL-Adolescent was developed via an iterative process involving focus groups and cognitive interviews with adolescents and consultations with experts to ensure content and face validity. After multiple rounds of revisions, the revised item bank included 12 functional HL items, 15 interactive HL items, and 9 critical HL items. The assessments were validated using Rasch Measurement Model, and item-level invariance was established across gender (boys vs. girls), ethnicity, and age groups (12–15-year-olds vs 16–18-year-olds). The final functional (6 items), interactive (10 items), and critical (7 items) HL assessments had good convergent validity with the Newest Vital Sign (NVS) and were positively related to applied HL behaviors (e.g., reading instructions before taking medicines). For the AAHL-Adult, the revised AAHL-Adolescent item bank was retained, and minor wording changes were made to 1 item (i.e., in a scenario related to school, the adult version asked about actions to help a child while the adolescent version asked about actions to help themselves) to make it more applicable to adults.

#### Newest Vital Sign (NVS)

Participants completed the NVS, a 6-item measure of their functional HL (24). For the NVS, participants were presented with an ice cream nutrition label and asked to answer 5 to 6 questions that assessed their reading and numeracy skills. Responses were scored, summed, and categorized into NVS-High Likelihood of Limited Literacy (0–1 correct), NVS-Possibility of Limited Literacy (2–3 correct), and NVS-Adequate Literacy (≥ 4 correct). The NVS has good internal consistency (Cronbach α = 0.76)(24) and summed scores were used to evaluate convergent validity with the AAHL-Adult.

#### Single-Item Literacy Scale (SILS)

The Single-Item Literacy Scale (SILS) is a 1-item perceptions-based measure of functional HL. Participants were asked, “How often do you need to have someone help you read instructions, pamphlets, or other written material from your doctor or pharmacy?” (25). Participants’ possible responses included never, rarely, sometimes, often, and always. Participants who endorsed never or rarely were categorized as SILS-Adequate HL, while all other responses were categorized as having difficulties with reading and writing health-related information (SILS-Inadequate HL). In their validation sample, Morris and colleagues (25) found that the measure had 54% sensitivity and 83% specificity in distinguishing people with limited reading ability.

#### HL Behaviors

Similar to the AAHL-Adolescent (21), participants were asked to endorse two behaviors including questions the truthfulness of health information found online and reads instructions before taking medicine, consistent with Sorenson and colleagues’ (2) description of the applied use aspect of HL. These items were used to establish criterion validity for the AAHL-Adult.

### Statistical Analyses

Winsteps (26) software was used to estimate Rasch measurement models. All other analyses were conducted in SPSS 28 (27).

### Rasch Analyses

#### Model Type.

The functional and interactive HL items had distinct correct and incorrect answers, therefore, Rasch dichotomous models were used to assess these scales. Several critical HL items included answers that spanned different levels of critical HL, so assessing the critical HL scale using the Rasch Partial Credit Model was most appropriate. Given that the composite model included all of the final items across the scales, including dichotomous and polytomous items, the Rasch Partial Credit Model was used to estimate this model.

#### Model Fit.

Person and item parameters were estimated using joint maximum likelihood estimation. Fit statistics (i.e., infit and outfit mean-squares and their standardized equivalents) were scrutinized for item and person parameters. Specifically, only if mean-squares were greater than 1.5, then their standardized statistics were reviewed, and if these standardized statistics were > 2 or <−2, then a significant misfit was indicated and the item was considered for removal (28). Given that outfit statistics are more sensitive than infit statistics to misalignment of person responses and item difficulties, more emphasis was put on outfit statistics. For each assessment, items were removed iteratively, starting with the most misfitting item (e.g., highest mean-square outfit misfit and standardized outfit statistic > 2). The models were re-estimated and fit statistics were reviewed after each item removal. As proposed by Linacre (28) and done in other studies (29, 30), person misfit considerations included removing one round of the most misfitting responses, re-estimating the model, and comparing the fit statistics of the modified and original responses. If the modified responses improved model fit, then the modified dataset was retained and final analyses were conducted on this dataset. However, if the removal of responses did not improve model fit, the original dataset with all responses was retained. Items with negative and very low point-measure correlations are indicative of the items not belonging to the scale (22) and were removed. Key assumptions of Rasch were assessed at each iteration of model estimation. Unidimensionality (do items assess a shared latent construct?) was established if the eigenvalue of the unexplained variance in the first contrast in the principal components analysis of the residuals was < 2 (22, 31). Local independence (are item responses statistically independent of each other?) was confirmed by comparing the Q_3_,* test statistic (i.e., maximum standardized residual correlation between a pair of items [Q_3,max_] - mean of all standardized residual correlations between item pairs [mean of Q_3_,]) to the critical values reported by Christensen and colleagues (32). Critical values for Q_3_,* test statistic at the 95th and 99th percentile are 0.26 and 0.31, respectively. To assess the monotonicity of the latent trait (are scores monotonically non-decreasing across the latent trait?) assumption, test characteristic curves were scrutinized to ensure they were monotonically ascending (33). As with other studies using Rasch, statistical findings and theoretical reasonings informed final decisions to retain or delete items (34). See Table 1 for item difficulties and fit statistics for the final assessments.

#### Reliability.

Item- and person-separation reliability were examined. For item-separation reliability, the goal is to have a good item difficulty range indicated by item-separation reliability statistics closer to 1. Regarding person-reliability, both Rasch person-reliability and classical test theory reliability statistics assume symmetry in ability. Wright (35) argued that symmetry in ability is rare in health-related research and proposed the Wright sample-independent person (test) reliability statistic to account for the lack of symmetry in samples. The Wright sample-independent person (test) reliability statistic is calculated after measure calibration (35) and used in place of Winsteps’ person reliability statistics. The 2-step procedure involves determining the number of strata across the scores and then using this to calculate the sample-independent reliability. Given the focus on a health topic, sample-independent reliability was appropriate for this study.

#### Invariance.

Uniform differential item functioning (DIF) was assessed for age groups, sex, race, ethnicity, and education. At least 100 participants per subgroup is recommended to detect DIF that is ≥ 0.5 logits and statistically significant (36). Due to small cell sizes, age was grouped into 25–29, 30–39, 40–49, 50–59, and ≥ 60 years to calculate DIF. However, the oldest group was still smaller than the recommended sample size (n = 93). For race, due to small cell sizes, Native American, Alaska Native, Hawaiian and Other Pacific Islander were combined into one group, however, this combined group was still smaller than recommended (n = 60). Similarly, <high school and high school graduate were combined due to the small cell size for < high school (n = 39). Some college and Associate’s degree were also combined, given the potential overlap in the number of years of education completed. DIF was assessed by asking whether the item has the same difficulty as the average difficulty across groups. For all DIF analyses, DIF size (≥ 0.64 logits) and significance thresholds were considered per the recommendations of Linacre (37).

#### Sample adequacy.

The sample size was appropriate for the Rasch model calculations as models can be estimated with 99% confidence within 0.5 logits with a minimum sample of 108 to 243 (38). For the Rasch Partial Credit Model, each response category surpassed the minimum requirement of 10 responses (39).

### Other Analyses

Descriptive statistics were calculated for the final AAHL-Adult. Pearson correlations between the summed scores of the NVS and the AAHL-Adult were computed to establish convergent validity (40). Point-biserial correlations between the dichotomized SILS and the assessments of HL were also computed to assess convergent validity. The NVS and SILS only measure functional HL; therefore, only moderate correlations with the interactive and critical HL assessments were expected. To assess criterion validity, logistic regressions predicting HL-related behaviors from AAHL-Adult after accounting for demographics (i.e., age, gender, race, ethnicity, education) were modeled. Receiver Operating Characteristic (ROC) curves (41) were estimated to determine the sensitivity (proportion of positive results that are true positives) and specificity (proportion of negative results that are true negatives) of AAHL-Adult assessments to predict NVS and SILS categories as well as the HL-related behaviors. Mean scores and categorizations across assessments were compared using independent t-tests and one-way analysis of variance. Where applicable, Bonferroni corrections were calculated. Effect sizes (the mean difference between z-scores across categories of the other assessments) were also computed. Lastly, crosstabs, chi-squares, and Cramer V tests were calculated to illustrate how categorizations across the assessments were related.

## Results

The sample included 2346 adults who were parents or caregivers of adolescents (Mean age = 43.02 years, SD = 8.41; ~63% women). Five participants identified as transgender, and one selected ‘prefer not to answer.’ Given the discrepancy in subsample sizes, gender-specific analyses were only conducted on persons who identified as binary (i.e., male, female). The largest racial group was White (~ 63%) and ~ 13% of the sample identified as of Hispanic or Latin origin. Approximately 53% of the sample had less than a Bachelor’s degree, and most participants had Adequate Literacy as assessed by the NVS (~ 55%) and SILS (~ 83%). See Table 2 for descriptive statistics.

### AAHL-Adult Functional Health Literacy (AAHL-Adult-FHL)

The AAHL-Adult-FHL item bank included 12 items. After the Rasch analyses, 5 items were removed, leaving a 7-item final AAHL-Adult-FHL assessment (see Appendix A). The final items assessed adults’ numeracy and reading comprehension skills using both a cafeteria menu and an over-the-counter-prescription drug label. Two items were removed due to outfit misfit. Three items were removed due to DIF on race. Removal of one round of the most misfitting person responses did not improve model fit, as such, the final model estimation used the original dataset. Point-measure correlations for the final 7 items were 0.43–0.61. Assumptions of unidimensionality (eigenvalue = 1.34), local independence (Q_3_,* test statistic=−0.14), and monotonicity were met. No DIF was detected for age group, gender, race, education, and ethnicity. Item separation reliability (1.00) was acceptable. The Wright sample-independent person (test) reliability statistic was 0.71, and the scores differentiated 1.6 distinct levels of performance: Inadequate (scores 0–4) and Adequate (scores 5–7).

AAHL-Adult-FHL scores (Mean = 5.50, SD = 1.43) differed by gender, age group, race, education, NVS, and SILS categories. Women had significantly higher AAHL-Adult-FHL scores than men (Mean difference [MD] = 0.56, p < 0.001). Regarding age, 50–59-year-olds had higher AAHL-Adult-FHL scores than 25–39-year-olds (MD = 0.41, p < 0.001) and 40–49-year-olds (MD = 0.24, p = 0.034). Adults who identified as Asian (MD = 0.36, p = 0.035) and Multiracial (MD = 0.53, p = 0.003) scored higher than those who identified as African American/Black. Participants who identified as Multiracial also scored higher than those who identified as White (MD = 0.42, p = 0.02). Regarding education, participants with a graduate degree had lower AAHL-Adult-FHL scores than those with ≤ high school diploma (MD=−0.33, p = 0.004), Associate’s/some college (MD=−0.73, p < 0.001), and a Bachelor’s degree (MD=−0.56, p < 0.001). Participants with an Associate’s/some college had higher AAHL-Adult-FHL scores than those with ≤ high school diploma (MD = 0.40, p < 0.001).

Participants who had NVS-Adequate Literacy had higher AAHL-Adult-FHL scores than those who had NVS-High Likelihood of Limited Literacy (MD = 2.20, p < 0.001) and NVS-Possibility of Limited Literacy (MD = 0.94, p < 0.001). Participants with NVS-Possibility of Limited Literacy had higher AAHL-Adult-FHL scores than those with NVS-High Likelihood of Limited Literacy (MD = 1.26, p < 0.001). Participants with SILS-Adequate HL had higher AAHL-Adult-FHL scores than those with SILS-Inadequate HL (MD = 1.58, p < 0.001).

Convergent validity was established with the NVS (*r* = 0.61, p < 0.001) and the SILS (*r* = 0.42, p < 0.001). The sensitivity of the AAHL-Adult-FHL to detect adults with NVS-Possibility of Limited Literacy (vs. NVS-High Likelihood of Limited Literacy) at the 4.5 cutoff score was 74.5%, and the specificity was 60% with an area under the ROC curve of 0.73 (CI: 0.69,0.76). The sensitivity of the AAHL-Adult-FHL to detect adults with NVS-Adequate Literacy (compared to NVS-High Likelihood of Limited Literacy and NVS-Possibility of Limited Literacy) at the 4.5 cutoff score was 94.9% and the specificity was 41.1%. The area under the ROC curve was 0.79 (CI: 0.78,0.81). For the SILS, the sensitivity of the AAHL-Adult-FHL to detect adults with SILS-Adequate HL at the 4.5 cutoff score was 85.3% and the specificity was 55.2% with an area under the ROC curve of 0.78 (CI:0.75,0.80).

Regarding criterion validity, AAHL-Adult-FHL was positively related to questioning the truthfulness of health information found online after adjusting for demographic covariates (OR = 1.19, CI:1.09,1.29, p < 0.001). The sensitivity and specificity of the AAHL-Adult-FHL assessment to detect adults who questioned the truthfulness of health information found online at the 4.5 cutoff score was 79.9% and 32.4%, respectively, with an area under the ROC curve of 0.58 (CI:0.54,0.62). AAHL-Adult-FHL was positively related to reading instructions before taking medicine (OR = 1.62, CI:1.37,1.91, p < 0.001). The sensitivity and specificity of the AAHL-Adult-FHL assessment to detect adults who read instructions before taking medicine at the 4.5 cutoff was 79.3% and 61.5%, respectively with an area under the ROC curve of 0.74 (CI:0.67,0.82).

Sensitivity and specificity at the cutoff scores are available in Table 3.

### AAHL-Adult Interactive Health Literacy (AAHL-Adult-IHL)

The AAHL-Adult-IHL item bank included 15 items. After the Rasch analyses, 5 items were removed, leaving a 10-item final AAHL-Adult-IHL assessment (see Appendix B). Similar to the adolescent interactive HL assessment, the AAHL-Adult-IHL tested adults’ skills for interacting with providers, multiple sources of contradictory information, and using knowledge to inform current behavior. Three items were removed due to low point-measure correlations. One item was removed due to high DIF on race and education, while another item was removed due to high DIF on race and age. Removal of one round of the most misfitting person responses improved model fit, as such, the final model estimation was completed using the adjusted dataset. Point-measure correlations for the final 10 items were 0.35–0.53. Assumptions of unidimensionality (eigenvalue = 1.26), local independence (Q_3_,* test statistic = 0.13), and monotonicity were met. No DIF was detected for gender, education, and ethnicity. However, DIF was detected for race for items ICHLD7 and ICHLD18. Compared to the average difficulty of all of the groups combined, ICHLD7 was more difficult (−0.91 logits, p < 0.001) and ICHLD18 was less difficult (0.84 logits, p < 0.001) for Asian respondents. ICHLD7 assesses decision-making about a second opinion and ICHLD18 assesses decision-making about help-seeking. The items were not deleted because the advantage of ICHLD18 compensates for the possible disadvantage caused by ICHLD7. Compared to the average of all groups combined, ICHLD8 was more difficult (−1.31 logits, p = 0.03) for respondents ≥ 60-years-old. In addition to the sample size being below the threshold to accurately detect DIF, ICHLD8 assesses navigating contradictory information (provider vs. social media post), DIF could reflect age-related trends in trusting misinformation online that is documented in the literature (42, 43) and a true disadvantage in interactive HL in this group. Hence, the item was not removed. Item separation reliability (1.00) was acceptable. The Wright sample-independent person (test) reliability statistic was 0.84 and the scores differentiated 2.3 distinct levels of performances: Inadequate (scores 0–4), Adequate (scores 5–8), and Proficient (scores 9–10).

AAHL-Adult-IHL scores (Mean = 6.31, SD = 1.99) differed by gender, age group, race, education, NVS, and SILS categories. Women had significantly higher AAHL-Adult-IHL scores than men (MD = 0.93, p < 0.001). Regarding age, 50–59-year-olds had higher AAHL-Adult-IHL scores than 25–39-year-olds (MD = 0.60, p < 0.001) and 40–49-year-olds (MD = 0.44, p = 0.001). Adults who identified as Multiracial scored higher than those who identified as African American/Black (MD = 0.67, p = 0.01) and White (MD = 0.79, p < 0.001). Adults with a graduate degree had lower AAHL-Adult-IHL scores than those with ≤ high school diploma (MD=−0.70, p < 0.001), Associate’s/some college (MD=−1.12, p < 0.001), and a Bachelor’s degree (MD=−0.68, p < 0.001). Adults with an Associate’s/some college had higher AAHL-Adult-IHL scores than those with ≤ high school diploma (MD = 0.42, p = 0.003) and a Bachelor’s degree (MD = 0.43, p < 0.001).

Adults who had NVS-Adequate Literacy had higher AAHL-Adult-IHL scores than those who had NVS-High Likelihood of Limited Literacy (MD = 2.66, p < 0.001) and NVS-Possibility of Limited Literacy (MD = 0.97, p < 0.001). Adults with NVS-Possibility of Limited Literacy had higher AAHL-Adult-IHL scores than those with NVS-High Likelihood of Limited Literacy (MD = 1.69, p < 0.001). Adults with SILS-Adequate HL had higher AAHL-Adult-IHL scores than those with SILS-Inadequate HL (MD = 2.21, p < 0.001).

Convergent validity was established with the NVS (*r* = 0.52, p < 0.001) and SILS (*r* = 0.42, p < 0.001). The sensitivity of the AAHL-Adult-IHL assessment to detect individuals with NVS-Possibility of Limited Literacy (vs. NVS-High Likelihood of Limited Literacy) at the 4.5 cutoff score was 82.3%, and the specificity was 49.4% with an area under the ROC curve of 0.70 (CI:0.67,0.74). When a cutoff score of 8.5 was applied, the sensitivity and specificity was 6.9% and 97%, respectively. The sensitivity of the AAHL-Adult-IHL to detect individuals with NVS-Adequate Literacy (compared to NVS-High Likelihood of Limited Literacy and NVS-Possibility of Limited Literacy) at the 4.5 cutoff was 96.6% and the specificity was 31.7%. At the 8.5 cutoff score, the sensitivity and specificity were 15.4% and 99.5%, respectively. The area under the ROC curve was 0.73 (CI: 0.71,0.75). For the SILS, the sensitivity of the AAHL-Adult-IHL assessment to detect individuals with SILS-Adequate HL at a 4.5 cutoff score was 90.2% and specificity of 47.5% with an area under the ROC curve of 0.77 (CI:0.74,0.79). At the 8.5 cutoff score, sensitivity and specificity were 12% and 97.6%, respectively.

Regarding criterion validity, AAHL-Adult-IHL was positively related to questioning the truthfulness of health information found online after adjusting for demographic covariates (OR = 1.16, CI:1.09,1.23, p < 0.001). The sensitivity and specificity of the AAHL-Adult-IHL to detect adults who questioned the truthfulness of health information found online at the 4.5 cutoff score were 85% and 26.6%, respectively, with an area under the ROC curve of 0.61 (CI:0.57,0.64). At the 8.5 cutoff score, the AAHL-Adult-IHL had a sensitivity and specificity of 11.3% and 96.1%, respectively. AAHL-Adult-IHL was positively related to reading instructions before taking medicine (OR = 1.59, CI:1.40,1.81, p < 0.001). The sensitivity and specificity of the AAHL-Adult-IHL to detect individuals who read instructions before taking medicine at the 4.5 cutoff score were 84.8% and 65.4%, respectively, with an area under the ROC curve of 0.82 (CI:0.77,0.88). At the cutoff score of 8.5, the sensitivity was 10.6% and the specificity was 100%.

### AAHL-Adult Critical Health Literacy (AAHL-Adult CHL)

The AAHL-Adult-CHL item bank included 9 items that assessed skills for health advocacy and engaging in decision-making where there are socioeconomic barriers. After evaluating the items using the Rasch Partial Credit Model, 7 items were retained for the final assessment (see Appendix C). Response options were ranked from not at all critical HL to collective advocacy skills, except for items CRHLD2 and CRHLD6 which were scored as incorrect or correct. Two items were removed due to DIF on education and race. Model fit did not improve when the most misfitting person responses were removed. Therefore, final Rasch analyses were conducted on the original dataset. Point-measure correlations for the final assessment were 0.42–0.63. Assumptions of unidimensionality (eigenvalue = 1.35), local independence (Q_3_,* test statistic= −0.14), and monotonicity were met. No DIF was detected for gender, education, age group, race, and ethnicity in the final model. Item separation reliability (1.00) was acceptable. The Wright sample-independent person (test) reliability statistic was 0.88, with the scores differentiating 2.7 two distinct levels of performance: Inadequate (scores 0–5), Adequate (scores 6–11), and Proficient (scores 12–15). Note that the scores ranged from 0–15 though only 7 items were retained. This is because with Rasch Partial Credit Models, each polytomous response option has a unique score that corresponds to the degree of correctness.

AAHL-Adult-CHL scores (Mean = 12.18, SD = 2.38) differed by gender, age group, education, NVS, and SILS categories. Women had significantly higher AAHL-Adult-CHL scores than men (MD = 1.44, p < 0.001). Regarding age, 50–59-year-olds had higher AAHL-Adult-CHL scores than 25–39-year-olds (MD = 0.93, p < 0.001) and 40–49-year-olds (MD = 0.65, p < 0.001). Adults ≥ 60-years-old also had higher AAHL-Adult-CHL scores than 25–39-year-olds (MD = 1.07, p < 0.001) and 40–49-year-olds (MD = 0.80, p = 0.011). Adults with a graduate degree had lower AAHL-Adult-CHL scores than those with ≤ high school diploma (MD=−1.13, p < 0.001), Associate’s/some college (MD=−1.55, p < 0.001), and a Bachelor’s degree (MD=−0.97, p < 0.001). Adults with an Associate’s/some college had higher AAHL-Adult-CHL scores than those with ≤ high school diploma (MD = 0.42, p = 0.016) and a Bachelor’s degree (MD = 0.58, p < 0.001).

Adults who had NVS-Adequate Literacy had higher AAHL-Adult-CHL scores than those who had NVS-High Likelihood of Limited Literacy (MD = 3.33, p < 0.001) and NVS-Possibility of Limited Literacy (MD = 1.34, p < 0.001). Adults with NVS-Possibility of Limited Literacy had higher AAHL-Adult-CHL scores than those with NVS-High Likelihood of Limited Literacy (MD = 1.99, p < 0.001). Adults with SILS-Adequate HL had higher AAHL-Adult-CHL scores than those with SILS-Inadequate HL (MD = 2.78, p < 0.001).

Convergent validity was established with the NVS (*r* = 0.55, p < 0.001) and SILS (*r* = 0.44, p < 0.001). The sensitivity and specificity of the AAHL-Adult-CHL to detect adults with NVS-Possibility of Limited Literacy (vs. NVS-High Likelihood of Limited Literacy) at the 5.5 cutoff score were 99.1% and 5.6%, respectively, with an area under the ROC curve of 0.73 (CI:0.70,0.76). At a cutoff score of 11.5, the sensitivity and specificity were 61% and 71.5%, respectively. The sensitivity and specificity of the AAHL-Adult-CHL to detect adults with NVS-Adequate Literacy (compared to NVS-High Likelihood of Limited Literacy and NVS-Possibility of Limited Literacy) at the 5.5 cutoff were 99.8% and 2.9%, respectively. At an 11.5 cutoff score, the sensitivity and specificity were 86% and 53.4%, respectively. The area under the ROC curve was 0.77 (CI: 0.75,0.79). The sensitivity and specificity of the AAHL-Adult-CHL assessment to detect adults with SILS-Adequate HL at the 5.5 cutoff score were 99.5% and 5.4%, respectively, with an area under the curve of 0.81 (CI:0.78,0.83). At the 11.5 cutoff score, the sensitivity and specificity were 76.3% and 71.4%, respectively.

Regarding criterion validity, AAHL-Adult-CHL was positively related to questioning the truthfulness of health information found online after adjusting for demographic covariates (OR = 1.11, CI:1.05,1.17, p < 0.001). The sensitivity of the AAHL-Adult-CHL to detect adults who questioned the truthfulness of health information found online at a 5.5 cutoff score was 98.6% and specificity was 1.4% with an area under the ROC curve of 0.59 (CI:0.55,0.62). At the 11.5 cutoff score, the AAHL-Adult-CHL had a sensitivity and specificity of 69.4% and 41.3%, respectively. AAHL-Adult-CHL was positively related to reading instructions before taking medicine (OR = 1.44, CI:1.30,1.60, p < 0.001). The sensitivity and specificity of the AAHL-Adult-CHL to detect individuals who read instructions before taking medicine at a 5.5 cutoff score were 98.7% and 7.7%, respectively, with an area under the ROC curve of 0.84 (CI:0.79,0.88). At the 11.5 cutoff score, the sensitivity and specificity were 69.2% and 80.8%, respectively.

### AAHL-Adult Composite HL (AAHL-Adult-Composite)

Using a Rasch Partial Credit Model, all of the items from the final AAHL-Adult-FHL, AAHL-Adult-IHL, and AAHL-Adult-CHL were fitted in a single model. Model fit statistics were acceptable. Point-measure correlations were 0.27–0.57. Assumptions of unidimensionality (eigenvalue = 1.41), local independence (Q_3_,* test statistic = 0.12), and monotonicity were met. No DIF was detected for gender, age group, education, and ethnicity. Similar to the IHL assessment, DIF was detected for race for items ICHLD7 and ICHLD18. Compared to the average difficulty of all of the groups combined, ICHLD7 was more difficult (−0.87 logits, p < .001), and ICHLD18 was less difficult (0.72 logits, p < .001) for Asian respondents. Item separation reliability (1.00) was acceptable. The Wright sample-independent person (test) reliability statistic was 0.95 with the scores differentiating 4.5 distinct levels of performance: Below Basic (scores 0–7), Basic (scores 8–15), Intermediate (scores 16–23), Proficient (scores 24–29), and Advanced (scores 30–32). The score range was larger than the number of items due to items with polytomous response options.

AAHL-Adult-Composite scores (Mean = 23.99, SD = 4.82) differed by gender, age group, race, education, NVS, and SILS categories. Women had significantly higher AAHL-Adult-Composite scores than men (MD = 2.91, p < 0.001). Regarding age, 50–59-year-olds had higher AAHL-Adult-Composite scores than 25–39-year-olds (MD = 1.91, p < 0.001) and 40–49-year-olds (MD = 1.35, p < 0.001). Adults ≥ 60-years-old also had higher AAHL-Adult-Composite scores than 25–39-year-olds (MD = 1.74, p = 0.005). Adults who identified as Multiracial scored higher than those who identified as African American/Black (MD = 1.70, p = 0.006) and White (MD = 1.78, p = 0.001). Adults with a graduate degree had lower AAHL-Adult-Composite scores than those with ≤ high school diploma (MD=−2.17, p < 0.001), Associate’s/some college (MD=−3.39, p < 0.001), and a Bachelor’s degree (MD=−2.23, p < 0.001). Adults with an Associate’s/some college had higher AAHL-Adult-Composite scores than those with ≤ high school diploma (MD = 1.22, p < 0.001) and a Bachelor’s degree (MD = 1.16, p < 0.001).

Adults with NVS-Adequate Literacy had higher AAHL-Adult-Composite scores than those with NVS-High Likelihood of Limited Literacy (MD = 8.17, p < 0.001) and NVS-Possibility of Limited Literacy (MD = 3.25, p < 0.001). Adults with NVS-Possibility of Limited Literacy had higher AAHL-Adult-Composite scores than those with NVS-High Likelihood of Limited Literacy (MD = 4.93, p < 0.001). Adults with SILS-Adequate HL had higher AAHL-Adult-Composite scores than those with SILS-Inadequate HL (MD = 6.55, p < 0.001).

Convergent validity was established with the NVS (*r* = 0.66, p < 0.001) and SILS (*r* = 0.51, p < 0.001). The sensitivity of the AAHL-Adult-Composite to detect adults with NVS-Possibility of Limited Literacy (vs. NVS-High Likelihood of Limited Literacy) at the 7.5 cutoff score was 100% and the specificity was 0.9%. At the 15.5 cutoff score, the sensitivity and specificity were 96.6% and 30.7%, respectively. At the 23.5 cutoff score, the sensitivity and specificity were 50.8% and 80.9%, respectively. At the 29.5 cutoff score, the sensitivity and specificity were 2.6% and 100%, respectively. The area under the ROC curve was 0.77 (CI: 0.74,0.80). The sensitivity of the AAHL-Adult-Composite to detect individuals with NVS-Adequate Literacy (compared to NVS-High Likelihood of Limited Literacy and NVS-Possibility of Limited Literacy) at the 7.5 cutoff score was 100% and the specificity was 0.4%. At the 15.5 cutoff score, the sensitivity and specificity were 99.7% and 15.4%, respectively. At the 23.5 cutoff score, the sensitivity and specificity were 86.6% and 63.1%, respectively. At the 29.5 cutoff score, the sensitivity and specificity were 12.5% and 99.9%, respectively. The area under the ROC curve was 0.83 (CI: 0.81,0.85). The sensitivity and specificity of the AAHL-Adult-Composite assessment to detect individuals with SILS-Adequate HL at the 7.5 cutoff score were 100% and 0.8%, respectively. At the 15.5 cutoff score, the sensitivity and specificity were 98% and 30.1%, respectively. At the 23.5 cutoff score, the sensitivity and specificity were 72.3% and 79%, respectively. At the 29.5 cutoff score, the sensitivity and specificity were 8.5% and 99.9%, respectively. The area under the ROC curve was 0.83 (CI: 0.81,0.86).

Regarding criterion validity, AAHL-Adult-Composite was positively related to questioning the truthfulness of health information found online after adjusting for demographic covariates (OR = 1.06, CI: 1.04,1.10, p < 0.001). The sensitivity and specificity of the AAHL-Adult-Composite to detect individuals who questioned the truthfulness of health information found online at the 7.5 cutoff score were 99.8% and 0%, respectively. At the 15.5 cutoff, the sensitivity and specificity were 93.1% and 6.7%, respectively. At the 23.5 cutoff, the sensitivity and specificity were 65.6% and 51.6%, respectively. At the 29.5 cutoff score, the sensitivity and specificity were 8% and 99.9%, respectively. The area under the ROC curve was 0.62 (CI: 0.58,0.65). AAHL-Adult-Composite was positively related to reading instructions before taking medicine (OR = 1.23, CI:1.17,1.30, p < 0.001). The sensitivity and specificity of the AAHL-Adult-Composite to detect adults who read instructions before taking medicine at the 7.5 cutoff score were 99.9% and 10%, respectively. At the 15.5 cutoff score, the sensitivity and specificity were 93.9% and 38.5%, respectively. At the 23.5 cutoff score, the sensitivity and specificity were 64.7% and 88.5%, respectively. At the 29.5 cutoff score, the sensitivity and specificity were 7% and 100%, respectively. The area under the ROC curve was 0.86 (CI: 0.81,0.90).

### Comparing AAHL-Adult-FHL, AAHL-Adult-IHL, AAHL-Adult-CHL, and AAHL-Adult-Composite Scores and Categories

Adults categorized as having Adequate functional HL in the AAHL-Adult-FHL had higher AAHL-Adult-IHL (*d* = 1.21), AAHL-Adult-CHL (*d* = 1.20), and AAHL-Adult-Composite (*d* = 2.04) scores than those categorized as Inadequate functional HL. Adults categorized as Proficient interactive HL in the AAHL-Adult IHL had higher AAHL-Adult-FHL (*d* = 1.55, 0.48), AAHL-Adult-CHL (*d* = 1.84, 0.55), and AAHL-Adult-Composite (*d* = 3.42, 1.36) scores than those categorized as Inadequate and Adequate interactive HL, respectively. Adults categorized as having Proficient critical HL in the AAHL-Adult-CHL had higher AAHL-Adult-FHL (*d* = 2.49, 1.02), AAHL-Adult-IHL (*d* = 2.81, 1.13), and AAHL-Adult-Composite (*d* = 6.26, 2.33) scores than those categorized as Inadequate and Adequate, respectively. Regarding AAHL-Adult-Composite, adults categorized as Advanced had higher AAHL-Adult-FHL scores than those categorized as Below Basic (*d* = 15.87), Basic (*d* = 4.43), Intermediate (*d*= 1.88), and Proficient (*d* = 0.94). Adults categorized as Advanced had higher AAHL-Adult-IHL scores than those categorized as Below Basic (*d* = 11.78), Basic (*d* = 5.81), Intermediate (*d*= 2.75), and Proficient (*d* = 1.62). Adults categorized as Advanced had higher AAHL-Adult-CHL scores than those categorized as Below Basic (*d* = 18.77), Basic (*d* = 5.51), Intermediate (*d*= 2.59), and Proficient (*d* = 1.15). All mean differences were significant at p ≤ 0.001. Table 4 shows the crosstabs based on AAHL-Adult HL categorization and all chi-squares and Cramer’s V tests were significant at the p < 0.001 level, suggesting that there is a relationship between the assessments when categories are used.

## Discussion

This study aimed to validate the AAHL, test-based assessments of adolescents’ functional, interactive, and critical HL, in an adult sample. Construct validity was established using Rasch Measurement models. All final assessments fit their Rasch models and met Rasch assumptions of monotonicity, unidimensionality, and local independence. When all assessments were modeled as a single scale, the items fit the Rasch Partial Credit Model and met Rasch assumptions. The assessments had good convergent validity with test-based and perceptions-based measures of functional HL and good criterion validity with HL-related behaviors.

The AAHL-Adult-FHL distinguished two categories, while both the AAHL-Adult-IHL and AAHL-Adult-CHL assessments distinguished three categories, and the AAHL-Adult-Composite distinguished five categories. The cutoff scores were determined using the Wright independent-sample reliability (test) statistic formula. The sensitivity and specificity of these cutoff scores to distinguish categories on the NVS and SILS, as well as individuals who engaged in HL behaviors were also considered. At the Adequate AAHL-Adult-IHL and AAHL-Adult-CHL cutoff scores, the assessments had higher sensitivity or better ability to identify respondents with NVS-Possibility of Limited Literacy, NVS-Adequate Literacy, SILS-Adequate HL, and who question the truthfulness of the information found online as well as read instructions before taking medicines. However, this was compromised by a lower ability to correctly identify respondents without the above characteristics. At Proficient AAHL-Adult-IHL and AAHL-Adult-CHL cutoff scores, the assessments had higher specificity and thus better ability to identify individuals who did not have NVS-Possibility of Limited Literacy, NVS-Adequate Literacy, SILS-Adequate HL, and who did not question the truthfulness of the information found online as well as read instructions before taking medicines. Regarding AAHL-Adult-FHL, the Adequate cutoff score showed good sensitivity across the NVS, SILS, and HL-related behaviors. However, acceptable specificity was only observed for NVS-Possibility of Limited Literacy and reading instructions before taking medicine. This assessment may be too short to be able to identify true negatives. Specificity improved at a cutoff score of 5.5; however, given the length of the assessment, the original cutoff score of 4.5 should be used, and the test be considered a rule-out rather than a rule-in test. Overall, the sensitivity and specificity analyses support the cutoff scores.

Nutbeam (12) argued that functional, interactive, and critical HL are distinct but related types of HL that require an increasingly complex set of skills, with functional HL being the simplest, followed by interactive HL, and then critical HL. This conceptualization was partially supported in this validation study. Specifically, the results support the relatedness of the three types of HL. The highest category on each assessment had the highest scores on the other assessments (e.g., Adequate AAHL-Adult-FHL category had the highest AAHL-Adult-IHL, AAHL-Adult-CHL, and AAHL-Adult-Composite scores). Non-parametric tests of the categorizations also confirmed linear relationships across the assessments. Contrary to Nutbeam’s (12) ordering of complexity of the HL types, the AAHL-Adult-IHL assessment appeared to be more difficult than the AAHL-Adult-CHL assessment. A look at Table 4 shows that more adults with Proficient AAHL-Adult-CHL were categorized as Adequate AAHL-Adult-IHL than Proficient AAHL-Adult-IHL. Further, only respondents categorized as Proficient AAHL-Adult-FHL and Proficient AAHL-Adult-CHL were categorized as Advanced Composite HL, while some respondents categorized as Adequate and Proficient AAHL-Adult-IHL were categorized as Advanced AAHL-Adult-Composite HL. A look at item difficulties for the AAHL-Adult Composite assessment in Table 1 supports that the 5 most difficult items out of the 24 items (20.8% and 50% of the AAHL-Adult-Composite and AAHL-Adult-IHL, respectively) were from the IHL assessment. Given these classifications, the AAHL-Adult-IHL assessment was the most challenging for the respondents in this validation sample.

Women scored higher than men across all assessments, consistent with several other studies (44–48). Participants 50–59-years-old scored higher on HL than younger age groups across assessments. Several studies have found that older age (> 65-years-old) is related to lower HL (1, 49); however, this sample was younger than those in these studies. Further, according to Sorensen and colleagues (2), HL develops across the lifespan, and more experience navigating health should lead to higher HL. Thus, there is likely a positive relationship between age and HL until the point in the lifespan where cognitive decline or generational differences in health care experience may contribute to a negative relationship between age and HL. Several studies have implicated that individuals who are racial and ethnic minorities have lower HL (1, 5, 48), however, participants who identified as Multiracial scored higher on the AAHL-Adult-FHL, AAHL-Adult-IHL, and AAHL-Adult-Composite than those identified as Black or White. Given the changing demographics of the U.S. population and the multiple social factors intertwined with race, racial differences in all three core qualities of HL should be explored further. Contrary to much of the research (1, 5, 50), individuals with a graduate degree had lower HL scores than those with less education. It is important to explore intersectionality (e.g., education and race) and consider other relevant variables (e.g., chronic disease status) when drawing conclusions about the HL/education relationship, especially given that interactive and critical HL may be more influenced by experiences navigating health decision-making rather than literacy.

To date, test-based interactive and critical HL measures have been limited (only 1 measure in Boston University Health Literacy Toolshed). In addition to providing test-based measures of adults’ interactive and critical HL, this study provides a single validated assessment that includes all three core qualities of HL described by Sorensen and colleagues (2) and Nutbeam (12), thus allowing for assessing and categorizing individuals’ core HL. Additionally, the existence of an adolescent version of the measure facilitates dyad studies assessing caregivers’ and adolescents’ HL.

A limitation of the study is that the use of a convenience, non-representative sample may limit the generalizability of the utility of the assessments. However, this is a minor concern given that Rasch models are sample-independent and less subject to sample bias than classical test theory. Another limitation is that the items may not be appropriate for all cultures. For example, the AAHL-Adult-FHL assessment requires reading a cafeteria menu and over-the-counter prescription label, individuals in cultures where these items are not common may have more difficulty answering related questions and be misclassified as having inadequate functional HL. Cultural appropriateness is an important consideration for using and/or adapting this measure. Relatedly, the sample size was appropriate for conducting Rasch analyses, however, it was insufficient for exploring DIF for age in years (vs age groups) and some racial groups. Future research should assess DIF for age in years, Alaskan Native, Native Americans, Native Hawaiian and Other Pacific Islanders, as well as other important demographic and social variables (e.g., federal poverty level, chronic illness status). The AAHL-Adult should also be included in longitudinal study designs to assess its predictive validity and time invariance.

The AAHL-Adult has both research and clinical utility. Many interventions targeting health behavior change employ strategies that require or intervene on functional, interactive, and critical HL but do not adequately assess HL in their evaluations. Including test-based assessments of HL in intervention batteries allow for evaluating HL as a moderator, mediator, and/or outcome and may help inform future intervention modifications to improve outcomes. Importantly, the AAHL-Adult is not subject to social desirability bias or people misestimating their abilities and competencies as is likely in perceptions-based instruments. Researchers and public health professionals may use the assessments to assess their intended populations to identify HL intervention needs and determine the suitability of their non-HL interventions. Regarding clinical utility, test-based assessments of HL may be used to identify adults who require assistance navigating their health care or inform how providers interact with patients.

## Conclusion

The finalized AAHL-Adult-FHL, AAHL-Adult-IHL, AAHL-Adult-CHL, and AAHL-Adult-Composite assessments met Rasch assumptions, had good model fit, and convergent and criterion validity. The AAHL-Adult-IHL and AAHL-Adult-CHL are the first test-based measures of interactive and critical HL validated in the U.S. The AAHL-Adult-Composite is also the first test-based assessment validated in the U.S. that measures all three core qualities of HL. These assessments have utility across multiple settings, including public health program planning and evaluation, intervention development and evaluation, and clinical settings. These assessments will also likely contribute significantly to how HL is studied in future studies.

## Supplementary Material

Supplement 1

## Figures and Tables

**Table 1:** Rasch item difficulties and fit statistics for AAHL-Adult

	Individual					Composite				
Item	Difficulty	SE	Outfit MNSQ	Outfit ZSTD	PMC	Difficulty	SE	Outfit MNSQ	Outfit ZSTD	PMC
**Functional HL**										
FHLD10	1.39	0.05	1.19	5.46	0.55	0.74	0.05	1.11	3.46	0.35
FHLD5	1.34	0.05	1.05	1.46	0.61	0.71	0.05	0.91	−2.91	0.47
FHLD6	0.49	0.06	0.89	−3.18	0.60	0.03	0.05	0.82	−4.29	0.48
FHLD9	0.09	0.06	1.18	3.81	0.46	−0.30	0.06	1.12	2.03	0.29
FHLD3	−0.474	0.07	0.74	−3.98	0.53	−1.02	0.07	0.71	−3.85	0.46
FHLD2	−1.21	0.08	1.02	0.19	0.40	−1.43	0.08	0.96	−0.39	0.28
FHLD11	−1.36	0.09	0.77	−2.39	0.43	−1.56	0.08	0.93	−0.58	0.33
**Interactive HL**										
ICHLD10	2.19	0.05	1.15	2.43	0.35	2.60	0.05	1.23	4.64	0.28
ICHLD18	1.09	0.05	0.97	−0.80	0.47	1.57	0.05	1.06	2.09	0.38
ICHLD13	0.90	0.05	1.10	3.20	0.43	1.38	0.05	1.16	6.05	0.33
ICHLD7	0.53	0.05	1.08	2.48	0.44	1.04	0.05	1.10	3.60	0.36
ICHLD2	0.48	0.05	0.93	−2.31	0.51	1.00	0.05	0.93	−2.63	0.47
ICHLD17	0.32	0.05	1.15	4.59	0.41	0.85	0.05	1.23	7.56	0.27
ICHLD14	−1.30	0.07	0.88	−1.66	0.48	−0.61	0.06	0.88	−1.79	0.40
ICHLD6	−1.31	0.07	0.90	−1.30	0.48	−0.61	0.06	0.97	−0.44	0.38
ICHLD16	−1.38	0.07	0.76	−3.42	0.53	−0.68	0.06	0.85	−2.34	0.45
ICHLD8	−1.51	0.07	0.74	−3.42	0.53	−0.80	0.06	0.74	−3.85	0.46
**Critical HL**										
CRHLD2	1.57	0.05	1.07	2.55	0.53	0.95	0.05	0.94	−2.08	0.45
CRHLPC11	0.50	0.04	0.91	−1.25	0.57	0.13	0.03	1.08	1.09	0.48
CRHLP4	0.19	0.04	0.97	−0.76	0.63	−0.25	0.04	0.92	−2.46	0.55
CRHLD6	−0.22	0.06	0.88	−1.77	0.42	−0.65	0.06	0.86	−2.15	0.38
CRHLP8	−0.59	0.04	1.18	5.81	0.54	−1.00	0.04	1.11	3.50	0.43
CRHLP7	−0.62	0.05	0.75	−4.04	0.52	−0.96	0.05	0.81	−2.83	0.48
CRHLP5	−0.83	0.04	0.67	−5.37	0.58	−1.15	0.04	0.71	−4.65	0.57

Notes. HL = health literacy; MNSQ = Mean-square; PMC = point-measure correlation; SE = standard error; ZSTD = standardized statistic

**Table 2:** Descriptives of the sample

Variable	n(%)	FHL M(SD)	F	IHL M(SD)	F	CHL M (SD)	F	Composite M (SD)	F
**Gender**			84.23**		121.20**		210.89**		209.15**
Men	867(37.1)	5.14(1.62)		5.73(2.26)		11.27(2.63)		22.15(5.52)	
Women	1473(62.9)	5.70(1.26)		6.65(1.73)		12.70(2.04)		25.06(3.99)	
Missing	6								
**Age (years)**			7.96**		9.15**		17.45**		16.26**
25–39	901(38.4)	5.34(1.49)		6.12(2.05)		11.86(2.43)		23.37(4.95)	
40–49	929(39.6)	5.52(1.44)		6.28(2.04)		12.14(2.47)		23.93(4.99)	
50–59	414(17.6)	5.76(1.21)		6.72(1.72)		12.79(1.97)		25.28(3.98)	
≥60	102(4.3)	5.55(1.36)		6.62(1.70)		12.94(1.70)		25.11(3.77)	
Missing	0								
**Hispanic/Latin origin**			2.03		0.60		2.28		0.57
No	2042(87.5)	5.52(1.43)		6.31(2.00)		12.22(2.37)		24.05(4.86)	
Yes	292(12.5)	5.39(1.41)		6.40(1.86)		11.99(2.30)		23.82(4.45)	
Missing	12								
**Race**			4.62*		6.60**		2.09		5.15**
AN/NA/NHOPI	60(2.6)	5.56(1.51)		6.88(1.41)		12.63(1.94)		25.07(3.87)	
Asian	206(8.8)	5.72(1.28)		6.55(1.86)		12.22(2.11)		24.50(4.17)	
Black	489(20.8)	5.37 (1.35)		6.32(1.89)		12.13(2.27)		23.87(4.44)	
White	1467(62.5)	5.47(1.48)		6.20(2.08)		12.12(2.49)		23.79(5.13)	
Multiracial	124(5.3)	5.89(1.04)		6.99(1.37)		12.68(1.94)		25.58(3.14)	
Missing	0								
**Education**			28.22**		31.99**		44.34**		50.97**
≤HS graduate	407(17.3)	5.36(1.37)		6.31(1.73)		12.29(2.23)		23.96(4.31)	
Associates/some college	848(36.1)	5.75(1.21)		6.72(1.67)		12.71(1.96)		25.18(3.76)	
Bachelor’s	626(26.7)	5.59(1.45)		6.29(2.10)		12.13(2.48)		24.03(5.09)	
Graduate degree	465(19.8)	5.03(1.68)		5.60(2.36)		11.16(2.71)		21.80(5.75)	
**Newest Vital Sign**			582.83**		372.97**		436.26**		768.61**
High likelihood limited literacy	437(20)	4.00(1.60)		4.45(2.40)		9.86(2.55)		18.31(5.31)	
Possibility of limited literacy	554(25.4)	5.25(1.21)		6.14(1.73)		11.85(2.12)		23.24(3.73)	
Adequate literacy	1189(54.7)	6.19(0.91)		7.11(2.01)		13.19(1.71)		26.49(2.88)	
Missing	166								
**Single-item HL Scale**			477.54**		484.01**		549.26**		806.23**
Inadequate HL	394(16.9)	4.20(1.65)		4.49(2.40)		9.88(2.57)		18.57(5.56)	
Adequate HL	1944(83.1)	5.77(1.21)		6.70(1.65)		12.66(2.02)		25.13(3.77)	
**AAHL-Adult-FHL**			4703.78**		558.75**		547.63**		1590.80**
Inadequate	492(21.6)	3.27(0.94)		4.62(2.26)		10.17(2.61)		18.08(4.82)	
Adequate	1786(78.4)	6.11(0.78)		6.77(1.63)		12.72(1.98)		25.62(3.32)	
Missing	68								
**AAHL-Adult-IHL**			304.52**		3117.53**		377.36**		1304.69**
Inadequate	372(16.4)	4.07(1.67)		2.84(1.26)		9.59(2.60)		16.51(4.44)	
Adequate	1668(73.3)	5.71(1.21)		6.69(1.03)		12.55(2.01)		24.95(3.14)	
Proficient	235(10.3)	6.27(0.88)		9.14(0.35)		13.61(1.28)		29.03(1.84)	
Missing	71								
**AAHL-Adult-CHL**			299.39**		370.58**		3430.45**		1608.30**
Inadequate	32(1.4)	3.25(1.65)		2.78(1.98)		4.44(0.67)		10.47(2.99)	
Adequate	695(30.6)	4.65(1.60)		5.02(2.17)		9.52(1.48)		19.20(4.08)	
Proficient	1547(68)	5.94(1.07)		6.97(1.48)		13.53(1.04)		26.43(2.54)	
Missing	72								
**AAHL-Adult-Composite**			560.02**		888.33**		1061.22**		3382.05**
Below Basic	4(0.2)	1.25(0.50)		0.75(0.96)		4.50(0.58)		6.50(5.77)	
Basic	152(6.7)	2.79(1.27)		2.13(1.51)		7.34(1.82)		12.26(2.10)	
Intermediate	672(29.7)	4.75(1.24)		5.19(1.45)		10.52(1.75)		20.46(2.25)	
Proficient	1275(56.3)	6.07(0.88)		7.10(1.14)		13.33(1.19)		26.49(1.63)	
Advanced	162(7.2)	6.86(0.35)		8.86(0.68)		14.65(0.54)		30.37(0.58)	
Missing	81								

AN/NA/NHOPI = Alaskan Native, Native American, Native Hawaiian/Other Pacific Islander; AAHL-Adult = Assessments of Adult Health Literacy; CHL = critical health literacy; FHL = functional health literacy; HL = health literacy; HS = high school; IHL = interactive health literacy; M = mean; SD = standard deviation

* p < 0.01

** p < 0.001

**Table 3:** Sensitivity and Specificity of AAHL-Adult assessments at cutoff scores

	Newest Vital Sign				Single-item Literacy Scale		Question truthfulness of online health information		Read instructions before taking medicine	
	Possibility of limited literacy		Adequate Literacy							
	Sensitivity	Specificity	Sensitivity	Specificity	Sensitivity	Specificity	Sensitivity	Specificity	Sensitivity	Specificity
**Functional HL**										
Adequate (> 4)	74.5	60	94.9	41.1	85.3	55.2	79.9	32.4	79.3	61.5
**Interactive HL**										
Adequate (> 4)	82.3	49.4	96.6	31.7	90.2	47.5	85	26.6	84.8	65.4
Proficient (> 8)	6.9	97	15.4	99.5	12	97.6	11.3	96.1	10.6	100%
**Critical HL**										
Adequate (> 5)	99.1	5.6	99.8	2.9	99.5	5.4	98.6	1.4	98.7	7.7
Proficient (> 11)	61	71.5	86	53.4	76.3	71.4	69.4	41.3	69.2	80.8
**Composite HL**										
Basic (> 7)	100	0.9	100	0.4	100	0.8	99.8	0	99.9	10
Intermediate (> 15)	96.6	30.7	99.7	15.4	98	30.1	93.1	6.7	93.9	38.5
Proficient (> 23)	50.8	80.9	86.6	63.1	72.3	79	65.6	51.6	67.4	88.5
Advanced (> 29)	2.6	100	12.5	99.9	8.5	99.9	8	99.9	7	100

HL = health literacy

**Table 4:** Cross tabs of categorizations of the sample based on AAHL-Adult cutoff scores

	Functional HL		Interactive HL			Critical HL			Composite HL				
	1^a^	2^b^	1^a^	2^b^	3^c^	1^a^	2^b^	3^c^	1^d^	2^e^	3^f^	4^c^	5^g^
	n (%)	n(%)	n(%)	n(%)		n(%)	n(%)		n(%)	n(%)	n(%)	n(%)	n(%)
**Functional**													
Inadequate (1)	--	--	218 (58.6)	265 (15.9)	7 (3)	26 (81.3)	298 (42.9)	164 (10.6)	4 (100)	139 (91.4)	275 (40.9)	70 (5.5)	0 (0)
Adequate (2)	--	--	154 (41.4)	1403 (84.1)	227 (97)	6 (18.8)	397 (57.1)	1382 (89.4)	0 (0)	13 (8.6)	397 (59.1)	1205 (94.5)	162 (100)
**Interactive**													
Inadequate (1)	218 (44.5)	154 (8.6)	--	--	--	25 (78.1)	258 (37.2)	87 (5.6)	4 (100)	141 (92.8)	204 (30.4)	21 (1.6)	0 (0)
Adequate (2)	265 (54.1)	1403 (78.6)	--	--	--	7 (21.9)	418 (60.3)	1242 (80.3)	0 (0)	11 (7.2)	465 (69.2)	1135 (89)	50 (30.9)
Proficient (3)	7 (1.4)	227 (12.7)	--	--	--	0 (0)	17 (2.5)	218 (14.1)	0 (0)	0 (0)	3 (0.4)	119 (9.3)	112 (69.1)
**Critical**													
Inadequate (1)	26 (5.3)	6 (0.3)	25 (6.8)	7 (0.4)	0 (0)	--	--	--	40 (100)	25 (16.4)	3 (0.4)	0(0)	0 (0)
Adequate (2)	298 (61.1)	397 (22.2)	258 (69.7)	418 (25.1)	17 (7.2)	--	--	--	0 (0)	127 (83.6)	470 (69.9)	96 (7.5)	0 (0)
Proficient (3)	164 (33.6)	1382 (77.4)	87 (23.5)	1242 (74.5)	218 (92.8)	--	--	--	0 (0)	0 (0)	199 (29.6)	1179 (92.5)	162 (100)
**Composite**													
Below Basic (1)	4 (0.8)	0 (0.0)	4 (1.1)	0 (0)	0 (0)	4 (12.5)	0 (0)	0(0)	--	--	--	--	--
Basic (2)	139 (28.5)	13 (0.7)	141 (38.1)	11 (0.7)	0 (0)	25 (78.1)	127 (18.3)	0(0)	--	--	--	--	--
Intermediate (3)	275 (56.4)	397 (22.3)	204 (55.1)	465 (28)	3 (1.3)	3 (9.4)	470 (67.8)	199 (12.9)	--	--	--	--	--
Proficient (4)	70 (14.3)	1205 (67.8)	21 (5.7)	1135 (68.3)	119 (50.9)	0 (0)	96 (13.9)	1179 (76.6)	--	--	--	--	--
Advanced (5)	0 (0)	162 (9.1)	0 (0)	50 (3)	112 (47.9)	0 (0)	0 (0)	162 (7.2)	--	--	--	--	--

HL = health literacy

a Inadequate health literacy

b Adequate health literacy

c Proficient health literacy

d Below Basic health literacy

e Basic health literacy

f Intermediate health literacy

g Advanced health literacy

Percentages are percent within column for each type of health literacy; All chi-squares and Cramer’s V were significant at p < 0.001; Contingency table for critical health literacy by AAHL-Adult composite had 5 cells (33%) with expected counts of less than 5.

## Abbreviations

AAHL-Adolescent: Assessments of adolescent health literacy
AAHL-Adult: Assessments of adult health literacy
CHL: Critical health literacy
CI: Confidence interval
DIF: Differential item function
FHL: Functional health literacy
HL: Health literacy
IHL: Interactive health literacy
MD: Mean difference
NVS: Newest vital sign
OR: Odds ratio
ROC: Receiver operating characteristic
SILS: Single-item literacy scale
