# Supplementary material for: Validation of the Assessments of Adult Health Literacy: A Rasch Measurement Model Approach
Source: Res Sq. 2023 Aug 9:rs.3.rs-3164944. Preprint. [Version 1] doi: 10.21203/rs.3.rs-3164944/v1 (PMC10441455; doi:10.21203/rs.3.rs-3164944/v1)
Supplement: Supplement 1 [file NIHPPrs3164944v1-supplement-1.pdf]

Supplementary Files

This is a list of supplementary files associated with this preprint. Click to download.

- AppendixAFHL.docx
- AppendixBIHL.docx
- AppendixCCHL.docx
